# Machine Embroidered Sensors for Limb Joint Movement-Monitoring Smart Clothing

**DOI:** 10.3390/s21030949

**Published:** 2021-02-01

**Authors:** Su Youn Park, Joo-Hyeon Lee

**Affiliations:** Department of Clothing &Textiles, Yonsei University, Seoul 03722, Korea; supark@yonsei.ac.kr

**Keywords:** machine embroidered sensor, fabric strain gauge, metal-fiber hybrid conductive yarn, limb joint motion monitoring, garment integrated sensing

## Abstract

In this study, a strain gauge sensor based on a change of contact or network structure between conductive materials was implemented using the handle-machine embroidery technique, and the variables (embroidery shape, embroidery distance, embroidery size, and implementation location) affecting its performance were studied. As a result of Experiment I on the structure of embroidery suitable for joint motion monitoring, the embroidery distance, rather than the embroidery size, was found to have a significant effect on the electric resistance changes caused by elongation. Based on the results of Experiment I, two types of zigzag embroideries, four types of embroideries with few contact points, and two types of embroideries with more contact points (all with short distances (2.0)) were selected for Experiment II (the dummy motion experiment). As a result of the dummy motion experiment, it was found that the locations of the suitable embroidered sensors for joint motion monitoring was the HJP (Hinge Joint Position) in the ‘types without a contact point’ (zigzag) and the LHJP (Lower Hinge Joint Position) in the ‘types with more contact points’. On the other hand, although there was no consistency among the ‘types with few contact points’, the resistance changes measured by the 2CP and 7CP embroidered sensors showed similar figures and patterns, and the HJP location was most suitable. The resistance changes measured by the 4CP and 6CP embroidered sensors exhibited no consistent patterns, but the LHJP locations were more suitable. These results indicate that the location of the HJP is suitable for measuring joint motion in the ‘type without a contact point’, and the location of the LHJP is suitable for measuring joint motion when the number of contact points exceeds a certain limit. Among them, the average resistance change of the 9CP sensor located at the LHJP was 40 Ω with the smallest standard deviation of less than 1, and it is thus considered to have the best performance among all the sensors.

## 1. Introduction

Recently, there has been a rapid increase in the demand for smart clothing that can monitor the movements of wearers without any constraints. Accordingly, flexible fabric-type motion monitoring sensors that are easy to apply in clothing are being developed. In addition, research on the materials and structure of sensors optimized for motion monitoring is being actively conducted. Most existing studies related to motion monitoring involve strain gauge sensors that utilize the principle of the piezo-resistance effect due to growth of the textile material. The strain gauge sensor developed by applying this principle requires a separate process of adhesion or attachment because it is made in the form of subsidiary materials, and there exists a measurement performance problem due to the unstable electrical resistance that occurs at this time and a limit of reproducibility as a sensor. There is also a durability limitation that is attributed to partial sensor detachment caused by repeated stretching.

Therefore, the aim of this study was the embroidering of conductive yarn into a garment and the examination of the effects of the major embroidery requirements (shape, distance, and size of the embroidery) on the performance of a limb joint motion monitoring sensor. In addition, after embroidery-based strain gauge sensors were implemented in the form of wearable platforms, the structure and implementation requirements of the machine embroidered sensors suitable for monitoring human limb joint motion were considered via experiments with a dummy model.

### 1.1. The Strain Gauge Sensor

Most previous studies related to strain gauge-type wearable motion sensors have been conducted with the aim of implementing sensors with a mixture of conductive materials and elastomers, such as polyurethane, to measure the length and resistance changes of sensors due to motion. The goal of previous studies was to implement a sensor with high sensitivity that has a large resistance change for a given length change by mixing conductive materials with flexible materials. Most of the conductive materials used were carbon-based materials, ICPs (inherently conducting polymers), silver, and silver nanowires, while urethane was used as an elastomer material with stretching properties.

Cochrane et al. (2010) developed a conductive polymer composite (CPC) with carbon black to investigate the effects of strain rate (from 10 to 1000 mm/min) under repeated elongation cycles. This study proved that the canopy fabric deformation during the critical inflation phase was successfully measured, and was found to be less than 9% [1]. Cho et al. (2020) studied an optimized design for joint-motion-sensing smart clothing based on the shape of the fabric sensors (both rectangular and boat-shaped) coated with single walled carbon nanotubes (SWCNTs) and the attachment location (knee and elbow joints or 4 cm below the knee and elbow joints). This confirmed that fabric sensors coated with SWCNTs are suitable for measuring joint motion in children [2]. Yamada et al. (2011) developed a strain gauge sensor with a structure in which thin SWCNT films are stacked on top of PDMS (polydimethylsiloxane) in parallel to maximize the piezo-resistance effect. This structure enabled the sensor to stretch up to 280%, and was found to be a high-sensitivity conductive sensor suitable for detecting a variety of motions ranging from delicate to large deformations, and which could be applied directly to clothes with fast sensing reactivity, low creep, and high durability [3].

Kim (2018) conducted a study on the optimized design of respiration monitoring smart clothing using sensors coated with multiwalled carbon nanotubes (MWCNTs) using a vacuum adsorption method on a polyurethane fabric surface. This study overcame the limitations of existing metallic materials and verified the possibility of using fabric materials coated with nonmetallic materials as strain gauge sensors [4]. Bilotti et al. (2010) developed a strain gauge sensor in the form of a string (strap) with a diameter of 3 mm by coating MWCNTs (2–3wt.%) on polyurethane fabric. In this case, the resistance of the material differed depending on the injection temperature conditions, with the resistance value increasing linearly from 5% to 50%, such that up to 15 stretch–relax cycles could be sensed [5]. Zhang et al. (2012) developed a strain gauge sensor that can sense up to 10% deformation by coating a composite material of thermoplastic polyurethane and MWCNTs (0.015wt.%) into spandex multifilament yarn (940 dtex). However, the performance was limited due to severe hysteresis and permanent deformation during recovery [6]. Zhang et al. (2013) developed a flexible film sensor (250 μm, 5 × 5 mm^2^) by coating MWCNTs (2wt.%) on spandex fabric, and confirmed that it could sense from 30% deformation for up to 10 cycles [7].

To develop strain gauge sensors for motion monitoring, Jang et al. (2019) compared two coating methods (brush coating and doctor blade coating) for the application of graphene to PU NWs (polyurethane nanowebs) and examined the surface, chemical, and mechanical properties. As the electrical resistance changed according to the amount of graphene coated on the PU NW, the two coating methods could be compared to determine the optimum coating conditions [8]. Bae et al. (2013) developed a rosette sensor that can sense up to 7.1% deformation with 75–80% transparency using graphene and attached it to transparent gloves to monitor finger motion [9]. Kim et al. (2011) compared the sensitivity of a CNT nanofiller strain sensor with a PU-woven strain gauge sensor coated with graphene nanofiller. They found that the sensitivity of the 3 wt.% graphene/epoxy strain gauge sensor was approximately four times greater than the sensitivity of the MWCNT/epoxy strain gauge sensor under the same condition [10].

Mi et al. (2017) developed strain gauge sensors for wearables that are useful for the overall monitoring of the human body, such as joint motion, heart rate, and respiration rate, by coating reduced graphene oxide (rGO) on the surface of a highly stretchable elastic blended fabric using plasma treatment, dip coating, and hydrothermal reduction. Thus, the requirements of strain gauge sensors for wearables with high sensitivity, excellent stability, and excellent repeatability were achieved [11].

Fan et al. (2012) coated polyaniline (PANI) on polyurethane fiber surfaces by in-situ chemical oxidative polymerization to develop a strain gauge sensor that was 1.5 times more sensitive than existing strain gauge sensors and less affected by hysteresis [12].

Lee et al. (2014) developed a thin film-type strain gauge sensor coated with silver nanoparticles and demonstrated that a linear resistance change rate could be achieved with a deformation of at least 10% to up to 25% [13].

### 1.2. Mechanisms for the Application of Strain Sensors to Wearables

According to prior studies, conventional strain gauge sensors are mostly made in the form of clothing accessories such as tape or string by coating and spraying conductive materials on stretchable fabric surfaces. To apply these accessory-type sensors to wearables, an additional attachment process using adhesive film or snap buttons is required, which limits accurate sensing. The conductive snap button, which is mainly used when attaching a strain gauge sensor to a wearable platform, is a kind of sensor-to-platform fastener. Snap buttons are attached to the wire of smart clothing without breaking the conductivity to make contact. The state of the snap button attached to the garment surface determines the stability of the contact between the wires on the sensor-snap button-platform, and an unstable contact often causes problems. For example, if the snap button is not correctly attached to the sensing position, the contact becomes unstable, adversely affecting the sensing reproducibility. The increase in the resistance of the snap button itself also causes noise in the measurement, which may result in limitations to the measurement performance. Repeated stretching can also cause durability problems due to the removal of a sensor or part of the sensor from the snap button [14].

Another limitation of strain gauge sensors coated with conductive material is induced cracking of the thinly coated conductive material after repeated strain cycling. The opening and closure of microcracks under deformation changes the total amount of conductive contact, which causes reproducibility and reliability problems for the strain gauge sensor due to repeated extension–relaxation.

One of the methods to overcome the limit of strain gauge sensors that use a mixture of flexible textile and conductive coating is to utilize the resistance change of the sensor according to the change in contact area between conductive materials. This is another principle of strain gauge sensing applicable to smart clothing, which utilizes the change in superposition of contacts between conductive materials by mechanical deformation. In other words, the change in the contact structure or network structure can change the superposition of contacts between conductive materials. To implement a strain gauge sensor that applies the principle of the change in contact superposition by stretching, there is a method of knitting conductive yarn to create a connected loop structure, and measuring the contact resistance according to changes in the contact structure or network structure. Strain gauge sensors manufactured by the knitting method minimize the problem of the strain gauge sensor using the flexibility of the textile material because it is built into the garment and does not require separate attachment or adhesion processes.

The conductive yarn-knitted strain gauge sensor has a ‘conductive loop contact structure’ and ‘conductive loop network structure’ depending on the loop connection structure. A form in which conductance is woven in the direction of the course with interlocking is referred to as a ‘conductive loop contact structure’, in which case the resistance increases when extended and the resistance decreases when relaxed. In the ‘conductive loop contact structure’, the contact pressure between neighboring conductive loops before extension has the highest value, and if the force pulling in the course direction is applied, the contact pressure is reduced because the contact between neighboring conductive loops is loosened by the tensile force. The strain gauge sensor with a ‘conductive loop contact structure’ has a proportional value of contact pressure and contact resistance, resulting in an increase in electrical resistance with the degree of elongation [15].

The shape in which conductive yarn knitted loops intertwine with knitting to form a network structure is called a ‘conductive loop network structure’, in which case the resistance decreases when elongated and the resistance increases when relaxed. To form a ‘conductive loop network structure’, conductive loops can be arranged only in the wale direction and connected in both the course and the wale directions simultaneously. In this structure, the contact resistance between the loops is reduced when the pulling force is applied because it strengthens the contact between the conductive loops by the tensile force. The strain gauge sensor with a ‘conductive loop network structure’ has a semiproportional value of contact pressure and contact resistance, and consequently, the electrical resistance decreases with the rate of elongation [16]. However, a strain gauge sensor with the ‘conductive loop network structure’ comprises a network of conductive yarn loops in all directions, resulting in yarn segment transfer during deformation, not only in the reduction of resistance due to extension, but also in the formation of other resistance relationships with respect to various deformation-induced changes [17].

In addition, to implement a strain gauge sensor that applies the principle of changing the contact superposition by deformation, there is not only a knitting method with conductive yarn, but also a method of embroidering the conductive yarn directly on the surface of the fabric. Strazdiene et al. (2007) implemented strain gauge sensors directly on the surface of flexible fabrics, such as interlock fabric and E-band, using stainless-steel yarn, to derive the optimized structural requirements for fabric sensors that measure fine deformations of 5–20%. As a result, the optimal requirements for fabric-type sensors, which can measure dynamic data in a fine extension range of 4–16%, were derived as crocheted chain-type embroidery over a 3 cm wide E-band attached with two layers of adhesive film. In this case, the contact resistance decreased with elongation because of the ‘conductive loop network structure’ [18,19].

Strain gauge sensors implemented by mechanical embroidery techniques using conductive yarn can be applied directly to the garment surface, so the size and implementation location of the sensor can be selected more freely than with the knitting technique. Compared to fabric-type sensors that require attachment using the stretchable properties of the textile material, they can be applied directly to the surface of the garment, and so have higher durability because there is no risk of detachment. They also have the advantage of there being no problem with cracking due to repeated expansion–relaxation, and the effect of hysteresis due to repeated expansion is not significant. Embroidered strain gauge sensors also minimize problems with measurement performance degradation and reproducibility due to unstable attachment. Nevertheless, there is insufficient research on strain gauge sensors that utilize the changing contact superposition due to stretching. In particular, given the lack of knowledge in this area, it is not enough to implement strain gauge sensors using machine embroidery techniques.

### 1.3. Sensor Material

A metal-fiber hybrid conductive yarn is a minimum unit of material for clothing with morphological stability, and is easily applied to a garment with flexibility while maintaining the conductivity of the metal. As the level of inherent resistance varies depending on the composition of each metal, the material can be selected for use and purpose, and the thread form can be processed in various ways to easily meet the demand for a measurement level suitable for the end use. Metal-fiber hybrid conductive yarn has various uses, such as weaving and the fabrication of clothing subsidiary materials such as tape. Due to these advantages, metal-fiber hybrid conductive yarn has traditionally been used as the main material in smart clothing design. On the other hand, metal-fiber hybrid conductive yarn has limitations for use as a material for clothing because of the lack of fiber-specific properties such as flexural durability, chemical safety, washability, wearability, and flexibility, needed to maintain the conductivity of the metal. Recently, metal-fiber hybrid conductive yarn, which has the properties of, flexibility, durability as a clothing material, and metal conductivity, has been developed and commercialized to the extent that machine embroidery is possible.

### 1.4. Principle of the Handle Embroidery Machine

A sewing machine operates using the intercomplex mechanism of two belts (2), (7), an upper driveshaft (4), lower driveshaft (8), and a vertical axis (5), which converts the rotation of the motor into repeated motion. The principle of stitch-ing by machine sewing follows a step-by-step process. First, the electric motor [1] is operated by pressing the pedal. Second, the operated electric motor rotates a belt (2) located between two disks. Third, the hand heel (3) in contact with the belt transmits the rotational action of the belt to the upper driveshaft (4). The upper driveshaft (4) is a self-rotating cylinder that raises and lowers a rod in the vertical axis (5) connected to the needle (6), and activates the second belt (7) connected to the lower driveshaft (8). The driveshafts at the top and bottom rotate parallel to each other and generate vertical axis movement (5), and the upper and lower threads meet through the downward and upward movement of the needle (6). Unlike hand needles, the machine needle (6) has a sharp point at the end of the needle hole, al-lowing only the upper thread to pass through the fabric without directly piercing the fabric. This causes the needle (6) to form a loop in the pushed thread as it moves up and down. This loop is fixed by hanging on a rotating under-case (9) and it meets with the lower thread to form a knot. Finally, a stitch is realized by pulling the upper thread up and tightening the knots on the fabric.

## 2. Research Methods

In this study, a strain gauge sensor based on a change of contact or network structure between conductive materials was implemented using the handle-machine embroidery technique, and the variables affecting its performance were studied. To understand the variables affecting the performance of the machine embroidered sensor with a commercialized silver-fiber hybrid yarn, two experiments were conducted in this study. First, the structure of embroidery-based strain gauge sensor according to the ‘embroidery shape’, ‘embroidery distance’, and ‘embroidery size’ was considered. Second, the embroidery-based strain gauge sensor selected in Experiment I was applied to an elbow-mounted platform worn on a dummy arm in order to study which implemented strain gauge sensor location is most advantageous for monitoring limb joint motion.

### 2.1. Experiment I: Preliminary Evaluation of the Influential Variables for Embroidered Sensors

#### 2.1.1. The Sensor Material and Fabrication

**Figure 1:**
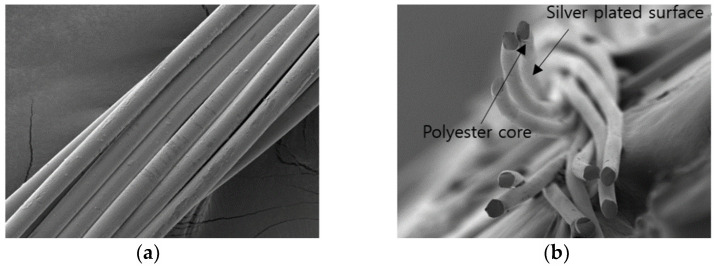
SEM (Scaning Electron Microscopy) images of the PE-silver plated yarn, IH#140D, (**a**) the surface and (**b**) the cross-section.

In this study, a limb joint motion monitoring sensor was implemented by applying the handle-machine embroidery technique mounted on a general sewing machine. The embroidery machine was a Brother ES-2400 model capable of implementing 59 types of stitching and embroidery. The material used as the substrate was a warp knit with a weight of 260/290 g/yd mixed with 91% polyester and 9% spandex. The commercially available silver-fiber hybrid yarn (IH#140D from Qingdao Tianyin Textile Technology Co., Ltd. in Beijing, China) used in the embroidery comprised 40 denier silver-plated polyester threads. Scanning electron microscopy (SEM) images show a total of 10 plies of silver-plated polyester yarns are twisted to form a single strand (Figure 1). In addition, an analysis of the heavy metal content using inductively coupled plasma-optical luminescence spectroscopy (ICP-OES) showed that silver comprised 113,000 mg/kg. The two types of sensors were produced by embroidery with both a single-strand and double-strand embroidery thread into a warp knit substrate. The single-strain-based sensor showed an electrical resistance of approximately 4.5 Ω/cm, and the double-strand based sensor exhibited an electrical resistance of approximately 2.1 Ω/cm.

#### 2.1.2. Experiment I Setup

The goal of Experiment I was to compare and analyze the performance of strain gauge sensors according to the structural conditions of the embroidery (embroidery shape, embroidery distance, and embroidery size), and to select the sensors to be used for Experiment II based on the results. This is because these three factors were assessed to affect the contact superposition in the embroidery, depending on the elongation–relaxation of the embroidery. The ‘embroidery shapes’ are divided into two types, the ‘type without a contact point’, which only spreads like a zigzag, and the ‘type with contact points’, including a ‘type with few contact points’ and a ‘type with more contact points’. In the ‘type without a contact point,’ three types of zigzag embroideries were selected. In the ‘type with contact points’, a total of six embroidery shapes were selected by selecting four types of embroideries with more than two contact points in a pattern of a unit for the ‘type with few contact points’ and two types of embroideries with more than nine contact points in a pattern of a unit for the ‘type with more contact points’.

**Figure 2:**
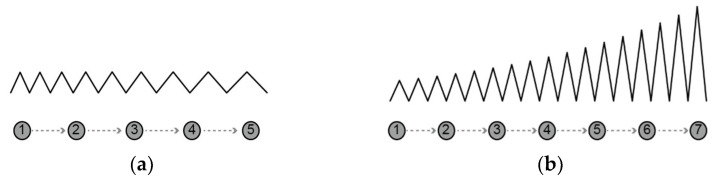
Embroidery distance adjustment (**a**) and embroidery size adjustment (**b**).

The ‘embroidery distance’ refers to the length of the straight-line distance between the starting point and the end point of a unit of embroidery, and in this study, only an ‘embroidery distance’ between 1.0 and 5.0 was conducted as determined by the available settings of the Brother ES-2400 sewing machine. The larger the ‘embroidery distance’ number (up to 5.0), the wider the spacing between the embroidery and the shorter the length of yarn used per unit length (Figure 2a). In order to measure the difference in resistance change according to the difference in the ‘embroidery distance’, a sensor corresponding to each of the two different embroideries, both short distance and long distance for each type, was produced and tested. For the production of narrow-spaced embroidery, a value of 2.0, which is less than the median number, was selected from the 1.0–5.0 spacing range of the Brother ES-2400 sewing machine, while a value of 4.0 was chosen for the production of wide-spaced embroidery.

The embroidery size refers to the width in the lateral direction of the embroidery, and was chosen from a scale of embroidery between 1.0 and 7.0 in this study, as determined by the Brother ES-2400 sewing machine. The larger the ‘embroidery size’ number (up to 7.0), the larger the size of the embroidery and the longer the length of yarn used per unit length (Figure 2b). In order to measure the difference in resistance change according to the difference in the ‘embroidery size’, small and large embroidery sensors were produced and tested for each type. For the production of small-sized embroidery, 3.0–4.0, which is less than the median number, was selected, while 5.0–7.0 was selected for the production of large-sized embroidery. The reason why the selected embroidery size variables are not fixed is that the embroidery size suitable for implementation with the flexible fabric varies. For this reason, there was a limit to controlling the embroidery size.

**Figure 3:**
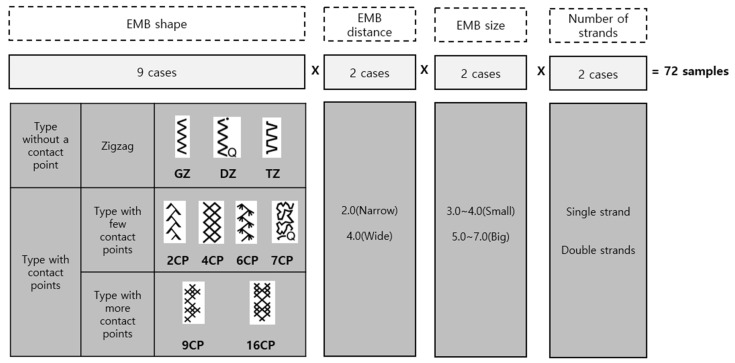
The 72 types of sensors.

A total of 72 types of embroidery (9 types of embroidery shape × 2 types of embroidery distance × 2 types of embroidery size × 2 different numbers of braided strands) were implemented to compare and analyze the resistance measured by varying the embroidery distance and size within the same embroidery shape. Nine types of embroidery were selected to compare the appropriate structural requirements of the joint motion monitoring strain gauge sensor according to ‘embroidery shape’, ‘embroidery distance’, and ‘embroidery size’, as shown in Figure 3. Five sets each of the 72 types of embroidered sensors were made with a 10 cm complete length using a sewing machine on the surface of a warp knit mixed with polyester/spandex. The implemented embroidery-based sensors were measured with a FLUKE 189 True RMS multimeter. The ‘resistance before extension’ of a 10 cm interval was measured, and the ‘resistance after extension’ was measured at a strain of 150% in the longitudinal direction. In order to verify the reliability of each embroidery-based sensor, the resistance of the five samples under the same condition was measured 10 times in total, and the difference between ‘resistance before extension’ and ‘resistance after extension’ was derived.

The shape dependence of the resistance change during elongation/relaxation for the zigzag-type embroidery was statistically analyzed for a given distance and size of the embroidery and the same number of braided strands. Three types of zigzag shapes were analyzed, with a Chi-square test conducted on the median of each group. If significant differences were found in the Chi-square test, a Bonferroni post hoc test was conducted to verify which groups had significant differences. In the Bonferroni post hoc test, the embroidery distance and size were controlled to analyze the differences in the embroidery shape. As a result of the Chi-square tests, three types of zigzag embroidery showed statistically significant differences between measurement performance that could be attributed to the embroidery shape for the same embroidery distance and size (*p*-value = 0.00 (<0.05)).

As a result of the Bonferroni post hoc test (Table 1), under all conditions with a single strand thread, the medians of ‘Decorative Zigzag (DZ)’ were higher than ‘General Zigzag (GZ)’ (*p*-value = 0.00 (<0.05). It was also shown that with the single strand thread, the medians of ‘Transformed Zigzag (TZ)’ were higher than GZ (*p*-value = 0.00 (<0.05). Between DZ and TZ, there was only a meaningful difference in the median of the electric resistance changes for an embroidery size of 3.0, with a single strand thread. For the embroidery distance setting of 2.0 with single-strand thread, the medians of DZ were higher than TZ (*p*-value = 0.00 (<0.05)). For the embroidery distance setting of 4.0 with single-strand thread, the medians of TZ were higher than DZ (*p*-value = 0.00 (<0.05)).

As a result of the Bonferroni post hoc test on the three types of zigzag embroidery with double-strand thread, the zigzag embroidery shape exhibited no consistent differences.

The results of the Bonferroni post hoc test on four types of ‘type with few contact points’ are shown in Table 2. For an embroidery distance of 4.0 with single-strand thread, regardless of the size of embroidery, the embroidery with seven contact points (7CP) was found to have the highest medians of the electric resistance changes compared to other embroidery shapes (*p*-value = 0.00 (<0.05)). For an embroidery distance of 2.0 with single-strand thread, regardless of the size of the embroidery, the embroidery with two contact points (2CP) was found to have the highest medians of the electric resistance changes relative to other embroidery shapes (*p*-value = 0.00 (<0.05)).

Regarding the results of Bonferroni post hoc test on four types of ‘type with few contact points’ with double strand thread, under all conditions, the 7CP embroidery exhibited the highest medians of the electric resistance changes compared to other embroidery shapes (*p*-value = 0.00 (<0.05)). The 2CP embroidery was found to have the lowest medians of the electric resistance changes for an embroidery distance of 4.0 (*p*-value = 0.00 (<0.05)). Overall, the results of ‘type with few contact points’ was found to be relatively consistent when it was implemented with single strand thread rather than double strand thread.

The results of the Kruskal–Wallis post hoc test on two types of ‘type with more contact points’ are shown in Table 3. For an embroidery distance setting of 2.0 with a single strand thread, the medians of embroidery with 16 contact points (16CP), regardless of the size of embroidery, was found to be higher than the embroidery with nine contact points (9CP) (*p*-value = 0.00 (<0.05)).

On the other hand, there was no statistically significant differences between the two types of embroidery with more contact points for an embroidery distance setting of 4.0 with single-strand thread. In addition, there were no significant differences between the two types of embroidery with more contact points using double-strand thread, regardless of the distance and size of the embroidery.

### 2.2. Experiment Ⅱ: Dummy Location Test of Strain Gauge Implementation

#### 2.2.1. Decision of Embroidery Type

Based on the results of Experiment I, the following sensors were selected for use in Experiment II. The embroidery structures selected for joint motion monitoring sensors were DZ and TZ at an embroidery distance setting of 2.0 (narrow spacing) and an embroidery size of 3.0 (small width) with single strand thread in the ‘type without a contact point’ (such as zigzag). In the ‘type with few contact points’, the 2CP, 4CP, 6CP, and 7CP embroidery were all selected with an embroidery distance of 2.0 (narrow spacing) and embroidery size of 3.5~4.0 (small width). The selected settings for the embroidery distance and size were the same because the resistance difference of the ‘type without a contact point’ and the ‘type with few contact points’ tended to be large for a narrow spacing and small width. For the ‘type with more contact points’ both the 9CP and 16CP were selected at an embroidery distance setting of 2.0 (narrow spacing) and size setting of 7.0 (large width). The embroidery selected from the three structures all showed a common tendency with the selection of a narrow spacing condition with an embroidery distance of 2.0.

#### 2.2.2. Dummy Model and Sensor Arm-Band

**Figure 4:**
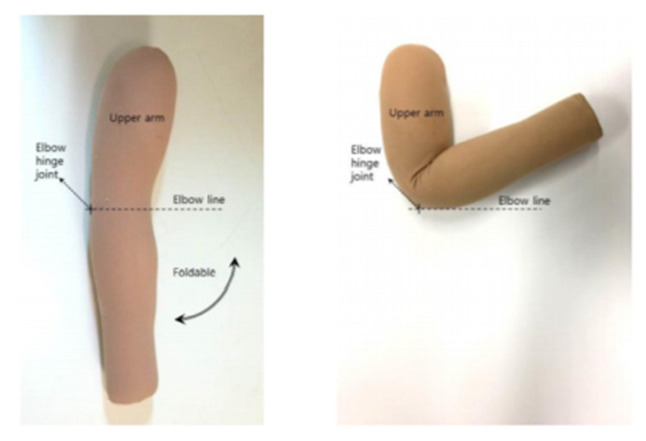
Foldable dummy arm.

An experiment was conducted using a dummy arm based on the standard Korean male size (arm length: 58.8 cm/armpit circumference: 42.9 cm/upper arm circumference: 30.1 cm/elbow circumference: 28.6 cm/wrist circumference: 16.4 cm) announced by the Korean Agency for Technology and Standards in 2010. The dummy is constructed with a fold around the elbow hinge joint to reflect the body’s movements as closely as possible (Figure 4).

**Figure 5:**
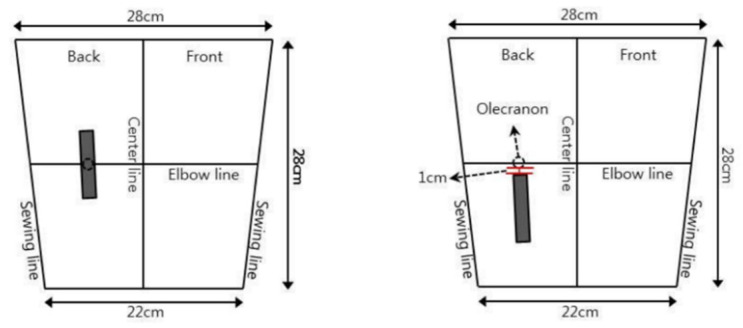
Sensor locations on the dummy arm.

The elbow-mounted platform worn on the dummy arm was made of high-stretch warp knit material with a weight of 260/290 g/yd mixed with 91% polyester and 9% spandex, and two locations to implement the machine embroidery-based sensors were selected (Figure 5). The center of the sensor located in the olecranon, the elbow hinge joint, and was called the ‘hinge joint position’ (HJP), and the center of the sensor located in the forearm distal to the olecranon was called the ‘lower hinge joint position’ (LHJP). The lower hinge joint position was set to be 1 cm under the actual olecranon of each subject, which was determined to be the best location for elbow movement sensing in a previous study.

#### 2.2.3. Elbow-Band for Limb Joint Movement Monitoring Dummy Test

##### Method and Procedure

**Figure 6:**
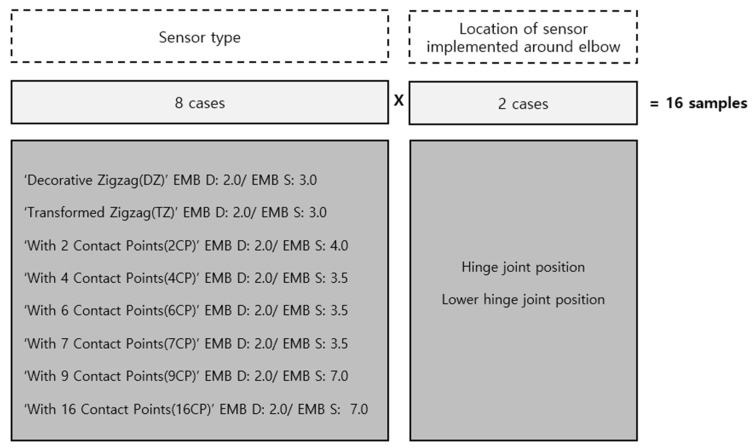
Eight different types of machine embroidered sensor for monitoring elbow motion.

Eight types of sensors suitable for monitoring limb joint motion, as selected by Experiment I (Figure 6), were implemented on the elbow-mounted platform worn on the dummy arm. In the next step, the performance of each sensor was compared and analyzed by measuring the electrical resistance changes by conducting Experiment II at a constant cyclic frequency (0.5 Hz) by fixing it to a dynamometer. The dynamometer is used to control the mediator variables that are affected by the deviation of the operating speed and the deviation of the operating angle.

**Figure 7:**
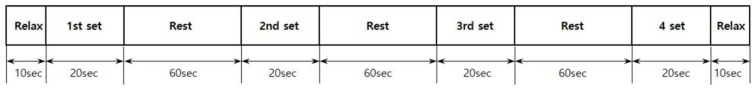
Protocol of Experiment II.

**Figure 8:**
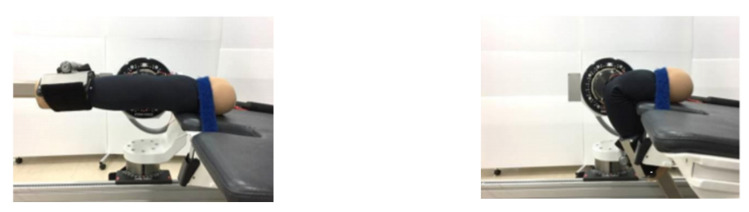
Dynamometer experiment.

Starting with the extended state of the dummy arm, one complete flexion and extension of the elbow joint was considered a single motion unit. One motion set comprised 10 motion units, and all motion sets were spaced 60 s apart. All wristlets were preconditioned for one motion set and a 60 s break. The timing of one motion unit was set to a normal motion speed of 0.5 Hz, which took 2 s for every single motion unit (Figure 7). The temperature and humidity of the laboratory were maintained at 25 °C and 55 ± 5%, respectively, during the experiment. The maximal angle of the flexed elbow joint was restricted to 120° (Floyd, R.T., 2007 [20]) using a dynamometer (CON-TREX MJ) (Figure 8).

#### 2.2.4. Experiment II Results

The electrical resistance changes due to joint motion (shaft-binary repetition) were measured across 16 combinations (8 types of sensor X 2 locations) of Experiment II. The data were evaluated with respect to two aspects: (1) the suitability for monitoring joint motion by comparing the average size of the resistance values measured in extension and flexion with the average size of the resistance changes due to the bending motion, and (2) the consistency of measurements according to a comparison of the graphed appearance of the signal morphology across 10 motion iterations.

The results of the dummy motion test showed that the average resistance at the relaxed state of the DZ sensor located at the HJP was 525 Ω, and that after flexion (after folding the arm) was 535 Ω, resulting in average resistance changes of 10 Ω (standard deviation: 2.45). The average electric resistance in the relaxed state of the DZ sensor located at the LHJP was 542 Ω, and that after flexion was 545 Ω, resulting in an average resistance change of 3 Ω (standard deviation: 5.09). This shows that the mean resistance changes of the DZ sensor at the HJP (10 Ω, standard deviation: 2.45) due to extension–flexion motion was greater than that measured at the LHJP (3 Ω, standard deviation: 5.09). In addition, the appearance of the graph exhibited by the DZ sensor located at the HJP tended to be more stable and consistent in terms of signal morphology than that of the DZ sensor located at the LJHP (Table 4).

The average resistance in the relaxed state of the TZ sensor located at the HJP was 636 Ω, and the average resistance after flexion was 647 Ω, resulting in an average resistance change of 11 Ω (standard deviation: 2.63). The average resistance in the relaxed state of the TZ sensor located at the LHJP was 535 Ω, and that after flexion was 541 Ω, resulting in an average resistance change of 6 Ω (standard deviation: 4.85). This shows that the mean resistance change of the TZ sensor at the HJP (11 Ω, standard deviation: 1.63) during the extension–flexion motion was greater than that at the LHJP (6 Ω, standard deviation: 4.85). In addition, the appearance of the graph exhibited by the TZ sensor located at the HJP tended to be more stable and consistent in terms of signal morphology than that measured by a TZ sensor located in the LJHP (Table 4).

These results indicate that the average of the resistance changes according to the extension–flexion motion measured in both types of zigzag sensors (DZ and TZ) was relatively larger in the HJP location. The implementation location of the zigzag embroidery more suitable for measuring elbow joint motion was determined to be the HJP. In addition, the appearance of the graphs representing the DZ and TZ sensor measurements, both implemented at the HJP location, tend to be more stable and consistent (in terms of signal morphology) than those corresponding to the sensors with the same structure implemented at the LHJP location. In terms of consistency of measurement, the HJP location is considered to be more suitable for measurements, and less influenced by noise (Table 4). However, in terms of the suitability for joint motion measurement, both zigzag types of embroidered sensors without contact points were considered unsuitable for detecting elbow movements with limited bending angles because the electric resistance changes due to the extension-flexion motion are relatively small compared to sensors with other structures. Meanwhile, the resistance changes measured by two types of embroidered zigzag sensors without a contact point, depending on the implementation location, were all found to be over 10 Ω at the HJP location, and under 6 Ω at the LHJP location, and similar measurement patterns were observed. This result indicates that the implementation location is a variable that has a greater impact on the results of the joint motion measurement than the structure of the zigzag embroidery.

Of the ‘type with few contact points’, the average resistance at the relaxed state of the 2CP embroidered sensor located at the HJP of the ‘type with few contact points’ was 749 Ω, and the that after flexion was 761 Ω, resulting in average changes of 12 Ω (standard deviation: 0.93). The average resistance at the relaxed state of the 2CP embroidered sensor located at the LHJP was 677 Ω, and that after flexion was 680 Ω, resulting in an average change of 3 Ω (standard deviation: 0.96). This showed that the mean resistance changes of the 2CP embroidered sensor located at the HJP (12 Ω, standard deviation: 0.93) during extension–flexion motion measurements was greater than that measured at the LHJP (3 Ω, standard deviation: 0.96). In addition, the appearance of the graph exhibited by the 2CP embroidered sensor located at both the HJP and LHJP tended to be stable and consistent in terms of signal morphology (Table 5).

The average resistance at the relaxed state of the 4CP embroidered sensor located at the HJP was 538 Ω, and that after flexion was 540 Ω, resulting in an average change of 2 Ω (standard deviation: 0.98). The average resistance at the relaxed state of the 4CP embroidered sensor located at the LHJP was 582 Ω, and that after flexion was 610 Ω, resulting in an average change of 28 Ω (standard deviation: 2.41). This showed that the mean resistance changes of the 4CP embroidered sensor located at the LHJP (28 Ω, standard deviation: 2.41) during the extension–flexion motion measurements was greater than that measured at the HJP (2 Ω, standard deviation: 0.98). In addition, the appearance of the graphs exhibited by the 4CP embroidered sensor located at both the HJP and LHJP tended to be stable and consistent in terms of signal morphology (Table 5).

The average resistance at the relaxed state of the 6CP embroidered sensor located at the HJP was 789 Ω, and that after flexion was 799 Ω, resulting in an average change of 10 Ω (standard deviation: 2.24). The average resistance at the relaxed state of the 6CP embroidered sensor located at the LHJP was 750 Ω, and that after flexion was 790 Ω, resulting in an average change of 40 Ω (standard deviation: 2.10). The mean resistance changes for the 6CP embroidered sensor located at the LHJP (40 Ω, standard deviation: 2.10) during the extension–flexion motion was greater than that measured at the HJP (10 Ω, standard deviation: 2.24). In addition, the appearance of the graphs of the 6CP embroidered sensor resistance located at the HJP and LHJP showed an unstable measurement pattern at the beginning of the operation, but with more than three repetitions of the operation, there was a tendency to become stable and consistent in terms of signal morphology (Table 5).

The average resistance at the relaxed state of the 7CP embroidered sensor located at the HJP was 1085 Ω, and that after flexion was 1105 Ω, resulting in an average change of 20 Ω (standard deviation: 2.3). The average resistance at the relaxed state of the 7CP embroidered sensor located at the LHJP was 1100 Ω, and that after flexion was 1103 Ω, resulting in an average change of approximately 3 Ω (standard deviation: 2.03). This showed that the mean resistance changes of the 7CP embroidered sensor located at the HJP (20 Ω, standard deviation: 2.3) during the extension–flexion motion was greater than the average change measured at the LHJP (3 Ω, standard deviation: 2.03). In addition, the appearance of the graphs corresponding to the 7CP embroidered sensor located at both the HJP and LHJP tended to be stable and consistent in terms of signal morphology (Table 5).

These results indicate that among the ‘types with few contact points’, the average resistance changes due to extension–flexion motion measured using the embroidered 2CP and 7CP sensors located at the HJP were greater than those measured at the LHJP (with resistance changes of less than 20 Ω). On the other hand, the average resistance changes due to extension–flexion motion measured using the embroidered 4CP and 6CP sensors located at the LHJP were greater than those measured at the HJP (with resistance changes of more than 20 Ω). Unlike the results of the ‘type without a contact point’ (zigzag) in which the HJP location was derived from the common tendency to be the most effective sensing location for elbow joint motion, the ‘type with few contact points’ did not exhibit a common tendency toward a single most effective sensing location. In other words, the ‘types with few contact points’ have the characteristic that random results are obtained with inconsistent measurement patterns regardless of the number of contact points, shape, and location of the embroidery, when the sensors are manufactured with the same material and length under controlled experimental conditions. These results indicate that in each case, the structure and implementation location are variables that affect the results of the joint motion measurement for the ‘type with few contact points.’

In terms of the suitability for joint motion measurement, both types of the embroidered 2CP and 7CP sensors, regardless of the implementation location, are considered unsuitable for detecting elbow movements with limited bending angles as the resistance changes due to extension–flexion motion are relatively small (i.e., resistance changes of less than 20 Ω) compared to sensors with other structures. On the other hand, in terms of the suitability for joint motion measurement, both types of the embroidered 4CP and 6CP sensors were considered suitable for detecting elbow movements with resistance changes of more than 20 Ω. Among the ‘types with few contact points’, the resistance changes exhibited by the embroidered 6CP sensor is the greatest (40 Ω), and is considered to be the most suitable for detailed measurements.

In terms of the consistency of the measurement signal morphology (as determined by the graphical appearance), the ‘type with few contact points’ was more stable, exhibiting consistent repeatability, than the ‘type without a contact point’ (zigzag). However, only the 6CP sensors were found to have unstable signals at the beginning of the operation.

Of the ‘type with more contact points’, the average resistance at the relaxed state of the 9CP embroidered sensor located at the HJP was 370 Ω, and that after flexion was 372 Ω, resulting in an average resistance change of 2 Ω (standard deviation: 0.96), while the average resistance at the relaxed state when located at the LHJP was 390 Ω, and that after flexion was 430 Ω, resulting in an average resistance change of 40 Ω (standard deviation: 0.83). This showed that the mean resistance changes of the 9CP embroidered sensor located at the LHJP (40 Ω, standard deviation: 0.83) due to extension–flexion motion was greater than that measured at the HJP (2 Ω, standard deviation: 0.96). In addition, the appearance of the graph exhibited by both 9CP embroidered sensors located at HJP and LHJP tended to be stable and consistent in terms of signal morphology (Table 6).

The average resistance at the relaxed state of the 16CP embroidered sensor located at the HJP was 671 Ω, and that after flexion was 690 Ω, resulting in an average change of 19 Ω (standard deviation: 1.48), while the average resistance at the relaxed state when located at the LHJP was 620 Ω, and that after flexion was 660 Ω, resulting in an average change of 40 Ω (standard deviation: 2.37). This showed that during extension–flexion motion, the mean resistance change of the 16CP embroidered sensor located at the LHJP (40 Ω, standard deviation: 2.37) was greater than that when located at the HJP (19 Ω, standard deviation: 1.48). In addition, the appearance of the graph exhibited by the 16CP embroidered sensors located at both the HJP and LHJP tended to be stable and consistent in terms of signal morphology (Table 6).

These results indicate that the 9CP and 16CP embroidered sensors of the ‘types with more contact points’ were considered suitable when the embroidery was implemented at the LHJP location, contrary to the results of experiments using the ‘type without a contact point’ (zigzag). While no consistent tendency was found for the appropriate sensor implementation location for the measurement of joint motion in the ‘type with few contact points’, this result verifies that for the ‘type with more contact points’, the location of embroidery implementation is more suited the LHJP. Thus, the implementation location of the embroidered sensors with many contact points is variable and affects the results of joint motion measurements. Among them, the average resistance change measured with the 9CP embroidered sensor at the HJP location was 2 Ω, the minimum value, but the change measured at the LHJP location was 40 Ω, which is the maximum value compared with the other embroidered structures. The results indicate that the implementation location for the 9CP embroidered sensor is a significant variable.

In terms of the suitability for joint motion monitoring, both types of the 9CP and 16CP embroidered sensors located at the LHJP measured average resistance changes of more than 40 Ω, which is greater than all the other sensors, and thus more suitable for monitoring fine movement (Table 7).

In terms of the consistency of measurement based on the signal morphology, as indicated by the appearance of graph, the ‘type with more contact points’ was more stable, with consistent repeatability, than the ‘type without a contact point’ (zigzag).

## 3. Conclusions

In this study, as a result of experiments on the structure of embroidery suitable for joint motion monitoring, the embroidery distance, rather than the embroidery size, was found to have a significant effect on the electric resistance changes caused by elongation.

For the ‘type without a contact point’ (zigzag), the conditions of short distance (2.0) and small size (3.0) for both DZ and TZ were considered suitable for measuring joint motion. In the case of the ‘types with few contact points’, a short distance (2.0) and small sizes (3.5–4.0) for the 2CP, 4CP, 6CP, and 7CP embroidered sensors were found to be suitable for joint motion measurement. For the ‘types with more contact points’, a short distance (2.0) and large size (7.0) for the 9CP and 16CP embroidered sensors were considered suitable for measuring joint motion, unlike those selected for the ‘type with few contact points’ and ‘type without a contact point’ (zigzag).

Based on the results of Experiment I, two types of zigzag embroideries, four types of embroideries with few contact points, and two types of embroideries with more contact points (all with short distances (2.0)) were selected for Experiment II (the dummy motion experiment). As a result of the dummy motion experiment, it was found that the locations of the suitable embroidered sensors for joint motion monitoring was the HJP in the ‘types without a contact point’ (zigzag) and the LHJP in the ‘types with more contact points’. On the other hand, although there was no consistency among the ‘types with few contact points’, the resistance changes measured by the 2CP and 7CP embroidered sensors showed similar figures and patterns, and the HJP location was most suitable. The resistance changes measured by the 4CP and 6CP embroidered sensors exhibited no consistent patterns, but the LHJP locations were more suitable. These results indicate that the location of the HJP is suitable for measuring joint motion in the ‘type without a contact point’, and the location of the LHJP is suitable for measuring joint motion when the number of contact points exceeds a certain limit.

In terms of conformity with the measurement of joint motion, the average resistance change exhibited by the 6CP, 9CP, and 16CP embroidered sensors located at the LHJP was 40 Ω, which was the most significant change measured, and was thus the most suitable machine embroidery-based sensor structure and the corresponding location.

Among them, the average resistance changes of the 9CP sensor located at the LHJP was 40 Ω with the smallest standard deviation of less than 1, and it is thus considered to have the best performance among all the sensors.

The consistency of the measured signal morphology (determined from inspection of the graph) was the most unstable in the sensors without a contact point; with a greater number of contact points, the results became stable and consistent. In this study, experiments were conducted on dummy arms to control individual differences, such as the effect of muscle function generated during extension–flexion movements, but this may differ from the actual arm motion of the human body. Therefore, subsequent studies are required to verify the results of this work. Further studies on the suitability of various other machine embroidery structures that can be implemented on flexible fabrics are also needed as the experiments herein were conducted only with limited types and conditions of mechanical embroidery.

## Figures and Tables

**Table 1:** The electric resistance changes for three types of zigzag embroidered sensor.

Electrode Type	Number of Strands	Condition: EMB Distance (EMB D) EMB Size (EMB S)	Median GZ (Ω)	Median DZ (Ω)	Median TZ (Ω)	Chi-Square Test	*p*-Value	Bonferroni Post Hoc Test
General Zigzag (GZ) vs. Decorative Zigzag (DZ) vs. Transformed Zigzag (TZ)	Single strand	EMB D: 2.0 EMB S: 3.0	30.00	50.20	34.60	102.43	0.00	DZ > TZ > GZ
		EMB D: 2.0 EMB S: 5.0~6.0	32.95	47.00	43.95	73.27	0.00	DZ > GZ TZ > GZ No consistency between DZ and TZ
		EMB D: 4.0 EMB S: 3.0	16.95	33.65	59.30	109.82	0.00	TZ > DZ > GZ
		EMB D: 4.0 EMB S: 5.0~6.0	22.70	35.65	36.95	71.15	0.00	DZ > GZ TZ > GZ No consistency between DZ and TZ
	Double Strands	EMB D: 2.0 EMB S: 3.0	5.10	11.10	10.65	86.29	0.00	DZ > GZ TZ > GZ No consistency between DZ and TZ
		EMB D: 2.0 EMB S: 5.0~6.0	13.45	12.90	11.65	21.07	0.00	DZ > TZ TZ > GZ No consistency between GZ and DZ
		EMB D: 4.0 EMB S: 3.0	14.40	25.20	5.80	116.54	0.00	DZ > GZ > TZ
		EMB D: 4.0 EMB S: 5.0~6.0	11.55	9.95	12.45	36.71	0.00	GZ > DZ TZ > DZ No consistency between GZ and TZ

**Table 2:** The electric resistance changes of four types of embroidered sensor with few contact points.

Electrode Type	Number of Strands	Condition: EMB Distance (EMB D) EMB Size (EMB S)	Median 2CP (Ω)	Median 4CP (Ω)	Median 6CP (Ω)	Median 7CP (Ω)	Chi-Square Test	*p*-Value	Bonferroni Post Hoc Test
2CP vs. 4CP vs. 6CP vs. 7CP	Single Strand	EMB D: 2.0 EMB S: 3.5~4.0	48.75	13.40	30.65	48.35	168.00	0.00	2CP > 6CP > 4CP 7CP > 6CP > 4CP No consistency between 2CP and 7CP
		EMB D: 2.0 EMB S: 7.0	55.95	9.35	17.95	40.10	186.55	0.00	2CP > 7CP > 6CP > 4CP
		EMB D: 4.0 EMB S: 3.5~4.0	25.35	22.05	24.30	36.05	138.73	0.00	7CP > 2CP > 4CP 7CP > 6CP > 4CP No consistency between 2CP and 6CP
		EMB D: 4.0 EMB S: 7.0	15.15	16.00	19.30	46.75	150.82	0.00	7CP > 6CP > 2CP 7CP > 6CP > 4CP No consistency between 2CP and 4CP
	Double Strands	EMB D: 2.0 EMB S: 3.5~4.0	6.95	4.50	7.45	29.25	157.06	0.00	7CP > 6CP > 4CP 7CP > 2CP > 4CP No consistency between 2CP and 6CP
		EMB D: 2.0 EMB S: 7.0	11.15	10.50	7.35	31.50	151.75	0.00	7CP > 2CP > 6CP 7CP > 4CP > 6CP No consistency between 2CP and 4CP
		EMB D: 4.0 EMB S: 3.5~4.0	6.25	13.90	7.90	22.50	173.88	0.00	7CP > 4CP > 6CP > 2CP
		EMB D: 4.0 EMB S: 7.0	4.30	10.25	6.30	26.70	170.29	0.00	7CP > 4CP > 6CP > 2CP

**Table 3:** The electric resistance changes of two types of embroidered sensor with more contact points.

Electrode Type	Number of Strands	Condition: EMB Distance (EMB D) EMB Size (EMB S)	Median 9CP (Ω)	Median 16CP (Ω)	Chi-Square Test	*p*-Value	Kruskal–Wallis Test
9CP vs. 16CP	Single strand	EMB D: 2.0 EMB S: 3.5	15.35	32.10	74.28	0.00	16CP > 9CP
		EMB D: 2.0 EMB S: 7.0	17.05	46.30	74.29	0.00	16CP > 9CP

**Table 4:** Electric resistance changes measured by two types of zigzag sensor during elbow joint location testing.

Sensor Type	Hinge Joint Point	Lower Hinge Joint Point
DZ (EMB distance: 2.0 EMB width: 3.0)	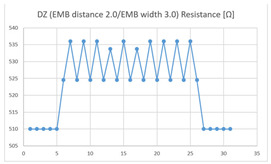	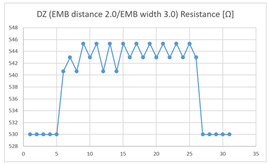
TZ (EMB distance: 2.0 EMB width: 3.0)	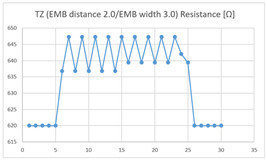	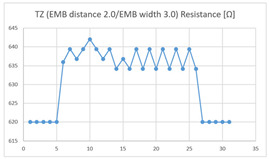

(Y axis: resistance (Ω); X-axis: time (s)).

**Table 5:** Electric resistance changes measured by four types of EMB sensor with few contact points during elbow joint location testing.

Sensor Type	Hinge Joint Point	Lower Hinge Joint Point
2CP (EMB distance: 2.0 EMB width: 4.0)	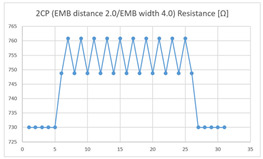	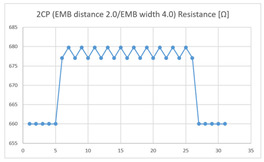
4CP (EMB distance: 2.0 EMB width: 3.5)	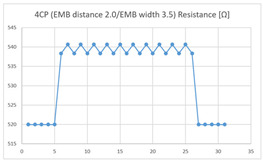	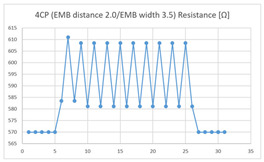
6CP (EMB distance: 2.0 EMB width: 3.5)	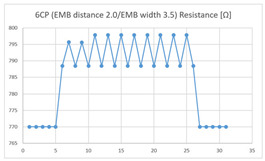	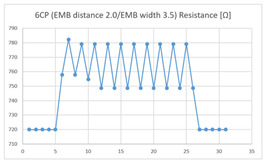
7CP (EMB distance: 2.0 EMB width: 3.5)	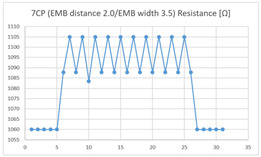	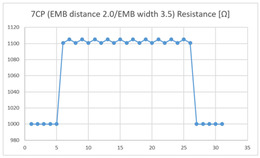

(Y axis: resistance (Ω); X-axis: time (s)).

**Table 6:** Electric resistance changes measured by two types of EMB sensor with many of contact points during elbow joint location testing.

Sensor Type	Hinge Joint Point	Lower Hinge Joint Point
9CP (EMB distance: 2.0 EMB width: 3.5)	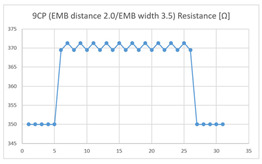	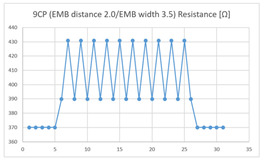
16CP (EMB distance: 2.0 EMB width: 3.5)	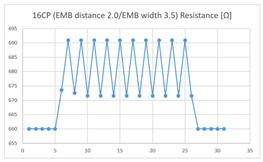	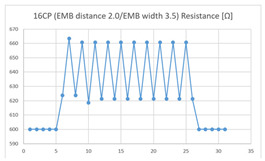

(Y axis: resistance (Ω); X-axis: time (s)).

**Table 7:** Resistance average and standard deviation across the 10-repeated motion.

Sensor Type	HJP				LHJP			
	Resistance Average in Extension	Resistance Average in Flexion	Electric Resistance Changes Averages in Extension–Flexion	St Dev	Resistance Average in Extension	Resistance Average in Flexion	Electric Resistance Changes Average in Extension–Flexion	St Dev
DZ	525 Ω	535 Ω	10 Ω	2.45	542 Ω	545 Ω	3 Ω	5.09
TZ	636 Ω	647 Ω	11 Ω	2.63	535 Ω	541 Ω	6 Ω	4.85
2CP	749 Ω	761 Ω	12 Ω	0.93	677 Ω	680 Ω	2 Ω	0.96
4CP	538 Ω	540 Ω	2 Ω	0.98	582 Ω	610 Ω	28 Ω	2.41
6CP	789 Ω	799 Ω	10 Ω	2.24	750 Ω	790 Ω	40 Ω	2.10
7CP	1085 Ω	1105 Ω	20 Ω	2.3	1100 Ω	1103 Ω	3 Ω	2.03
9CP	370 Ω	372 Ω	2 Ω	0.96	390 Ω	430 Ω	40 Ω	0.83
16CP	671 Ω	690 Ω	19 Ω	1.48	620 Ω	660 Ω	40 Ω	2.37

(Light-shaded: electric resistance changes average during extension-flexion > 20 Ω; dark-shaded: electric resistance changes average during extension-flexion ≥ 40 Ω).
